# Anti-NMDAR encephalitis presenting with persistent fever and meningitis and responding well to distinctive individualized first-line treatment: A case report

**DOI:** 10.1097/MD.0000000000040803

**Published:** 2024-12-06

**Authors:** Xin Jin, Jianhua Zhuang, Jin Xu

**Affiliations:** a Department of Neurology, Second Affiliated Hospital of Naval Medical University, Shanghai Changzheng Hospital, Shanghai, China.

**Keywords:** autoimmune encephalitis, case report, intravenous immunoglobulin, meningoencephalitis, seizures

## Abstract

**Rationale:**

Anti-N-methyl-D-aspartate receptor (NMDAR) encephalitis is an autoimmune encephalitis characterized by diverse neurological and psychiatric symptoms. It predominantly affects young women, particularly those with ovarian teratomas. However, cases without teratomas are also commonly reported, often exhibiting poorer treatment responses and higher relapse rates. Persistent fever and signs of meningitis are rare in such cases. Diagnosis is confirmed through the detection of anti-NMDAR antibodies.

**Patient concerns:**

A 15-year-old female presented with episodic loss of consciousness, fever, nuchal rigidity, limb convulsions, and psychiatric symptoms following a stressful exam period. Initial symptomatic treatments were ineffective.

**Diagnosis:**

The diagnosis of anti-NMDAR encephalitis was confirmed through lumbar puncture, brain imaging, and the detection of anti-NMDAR antibodies in cerebrospinal fluid and serum.

**Interventions:**

The patient received tailored first-line therapy, including high-dose methylprednisolone and 3 courses of intravenous immunoglobulin (IVIG).

**Outcomes:**

The patient exhibited significant clinical improvement, with a reduction in seizure frequency and eventual complete seizure control. Body temperature normalized, and follow-up showed progressive recovery in cognitive and motor functions.

**Lessons:**

This case highlights the importance of early diagnosis and individualized treatment in anti-NMDAR encephalitis. Repeated IVIG courses proved effective, underscoring the need for personalized treatment plans in managing this condition. Persistent fever and signs of meningitis were rare and contributed to the diagnostic challenge, highlighting the clinical complexity of this case.

## 1. Introduction

Anti-N-methyl-D-aspartate receptor (NMDAR) encephalitis, first reported in 2007,^[1]^ is an autoimmune disorder caused by auto-IgG antibodies targeting the GluN1 subunit of the NMDAR. These antibodies bind to the receptor, inducing internalization, crosslinking, and extracellular glutamate accumulation.^[2–4]^ This process leads to reversible synaptic alterations, reducing neuronal excitability and causing neurological symptoms.^[5]^ Crosslinking refers to the process where antibodies cause multiple receptors to bind together, resulting in their internalization and functional inhibition.^[6]^ This neuronal dysfunction, mediated by humoral immune mechanisms, is often effectively treated with immunotherapy.^[7]^ While anti-NMDAR encephalitis is frequently associated with ovarian teratomas, approximately 40% to 70% of cases in women of childbearing age occur without detectable teratomas.^[8]^ Diagnosis is typically established by detecting NMDAR antibodies in cerebrospinal fluid (CSF), following the widely used Graus and Dalmau criteria (2016).^[9]^

For patients with ovarian teratomas, early tumor resection is recommended, as it can significantly improve clinical outcomes.^[10–12]^ In non-tumor cases, such as the present case, the disease mechanisms primarily involve antibody-mediated synaptic disruption. Viral infections, particularly herpes simplex virus, are considered potential triggers. Additionally, broader immune dysregulation, including lymphocytic pleocytosis observed in CSF, may also contribute to the development of the disease.^[13]^ The clinical course of anti-NMDAR encephalitis typically peaks within weeks and primarily affects females, with common symptoms including psychiatric abnormalities, seizures, memory loss, and autonomic dysfunction. Less commonly, patients may present with meningitis, increased intracranial pressure, and neck rigidity.^[14]^ In non-tumor cases, the symptoms tend to be more severe and may require aggressive immunotherapy, including both first-line and second-line treatments.^[7]^ Immunotherapy options include corticosteroids, intravenous immunoglobulin (IVIG), plasma exchange, and long-term immunosuppressive therapies. However, there are no standardized guidelines for selecting or timing these treatments, emphasizing the importance of tailoring the treatment regimen to the individual patient’s needs.^[15]^

## 2. Case report

**Figure 1.**
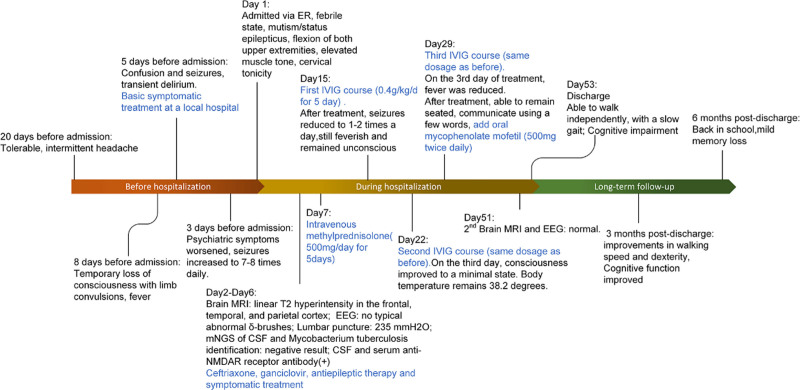
Patient clinical course timeline.

In July 2022, a 15-year-old girl was hospitalized with episodic confusion, limb twitching, and an 8-day fever (Fig. 1). One month before starting school, she stayed at her aunt’s countryside home after completing her high school entrance exam. She experienced intermittent, tolerable headaches 20 days before admission, without recorded fever. In the initial 3 days, she had 2 episodes of 1-minute loss of consciousness with limb convulsions. Subsequently, episodes of uncontrolled shouting occurred 2 to 3 times per day, with each episode lasting over ten seconds. Despite receiving symptomatic treatment at a local hospital, her psychiatric symptoms worsened, hindering communication with her family. Seizure frequency increased to 7 to 8 times daily, prompting immediate admission to our hospital’s intensive care unit for further treatment. Upon admission, she presented with a persistent fever. Neurological examination revealed mutism, flexion of both upper extremities, elevated muscle tone, cervical rigidity, and an inability to touch her chin to her chest. The impairments were rated using the modified Rankin Scale (mRS) and the Glasgow Coma Scale (GCS). The mRS^[16]^ is a 6-point single-item scale ranging from 0 to 5, designed to assess the degree of disability or dependence in the daily activities of individuals after a stroke or other neurological disability. Due to the patient’s comatose state, the mRS score of the young girl was 5, indicating severe disability. The GCS^[17]^ is used to evaluate the level of impaired consciousness in response to defined stimuli, encompassing 3 components: best eye response, best verbal response, and best motor response. Scores range from 3 to 15, with lower scores reflecting more severe levels of unconsciousness. The young girl’s GCS score was 6, indicating a significantly reduced level of consciousness. The most prominent symptoms contributing to this score were her inability to produce sounds, opening her eyes only in response to pain, and displaying abnormal flexion to painful stimuli. Brain magnetic resonance imaging (MRI) showed linear T2 hyperintensity in the frontal, temporal, and parietal cortex. Blood tests and virus tests showed no significant abnormalities. Serum tumor markers, chest CT, whole abdomen ultrasound, reproductive system ultrasound, and pelvic MRI (plain and enhanced) did not show evidence of a tumor. Effective antiepileptic therapy had been initiated before the examination, rendering the electroencephalogram (EEG) devoid of typical abnormal delta-brushes and epileptic waves. A lumbar puncture showed elevated cerebrospinal fluid pressure at 235 mmH2O (normal range: 80–180), but other biochemical tests, including IgG, leukocyte count, protein levels, and glucose levels, were unremarkable.

Considering the persistent fever, seizures, psychiatric symptoms, meningeal involvement on physical examination, and parenchymal and cortical involvement on brain MRI, meningeal and cortical encephalitis due to pathogenic infection was suspected. Despite 6 days of ceftriaxone antibacterial intervention, ganciclovir antiviral measures, antiepileptic therapy, and symptomatic treatment, the patient’s clinical condition did not improve, and body temperature remained between 37.5 and 38.3 °C. Due to the poor response to these treatments, high cerebrospinal fluid pressure, and atypical clinical presentation, tuberculosis infection could not be excluded. Subsequently, metagenomic next-generation sequencing (mNGS) of the patient’s cerebrospinal fluid identified approximately 17,500 pathogenic infections from various microorganisms, including bacteria, fungi, viruses, and parasites. No suspected pathogenic bacteria or Mycobacterium tuberculosis were found. Consequently, the trial of levofloxacin as an antituberculosis regimen was discontinued.

**Figure 2.**
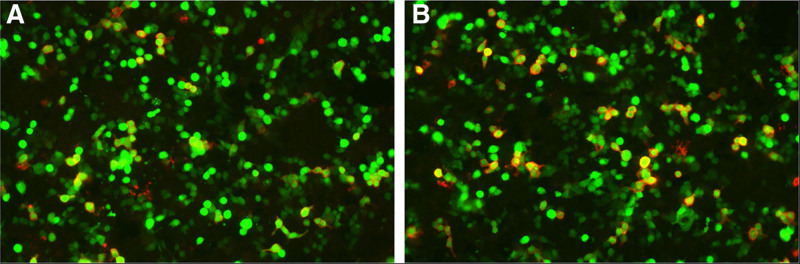
Anti-NMDAR antibody titer of serum 1:10+ positive fluorescence image (A). Anti-NMDAR antibody titer 1:1++ positive fluorescence image of cerebrospinal fluid (B). NMDAR = N-methyl-D-aspartate receptor.

Finally, the cell-based assay (CBA) results revealed the presence of anti-NMDAR antibodies in the patient. The serum antibody titer was 1:10 + (positive, high fluorescence intensity), and the cerebrospinal fluid antibody titer was 1:1++ (positive, high fluorescence intensity), with initial dilution gradients of 1:10 and 1:1, respectively (Fig. 2). The number of “+” indicates fluorescence intensity, with more “+” indicating higher antibody levels. The patient was diagnosed with anti-NMDAR encephalitis on the 7th day of admission, high-dose methylprednisolone at 500 mg/day was administered, but symptoms showed limited improvement after 5 days. After 2 days of observation with persistent fever (38°C) and 4 to 5 daily seizures, IVIG therapy was initiated at 24 g/day (0.4 g/kg/day) for 5 days. After IVIG treatment, seizures reduced to 1 to 2 times daily, but the patient remained febrile and comatose between seizures. A second course of IVIG was initiated due to the absence of improvement after 3 days of observation. By day 3 of the second IVIG session, the patient’s consciousness improved and returned to a minimally conscious state. However, her condition remained severe, with her eyes unable to open. She could only perform basic movements, such as gently moving her eyelids and raising a finger, upon request. Her body temperature remained at 38.2°C. Two days posttreatment, the patient’s symptoms remained unchanged. This situation was reminiscent of a plateau phase, where residual symptoms persisted without further improvement, similar to the initial IVIG treatment. Moreover, she continued to experience fever. At this juncture, we considered the possibility that the patient’s symptom relief might not be solely due to the effects of steroids, as the likelihood of steroids causing fluctuations in symptom relief (waves of improvement and plateau phases) over time was relatively low. We surmised that the patient exhibited an exceptionally positive response to IVIG, given the cumulative dosage. This suggests that the patient continues to benefit from the therapeutic effects of IVIG.

**Figure 3.**
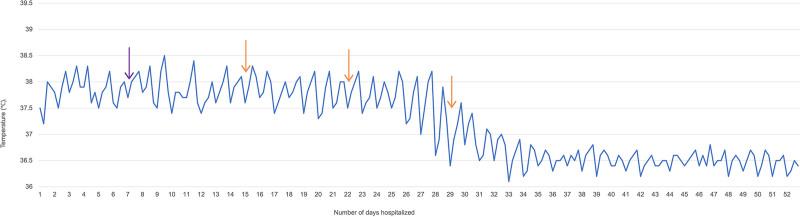
Temperature chart indicating fever trend in degree Celsius (°C). Purple arrow: Application of high-dose methylprednisolone Orange arrow: Three courses of IVIG use. IVIG = intravenous immunoglobulin.

Upon the fervent request of the patient’s mother, we meticulously conveyed the potential risks associated with the medication. Nevertheless, the family insisted on the third IVIG course. After comprehensive consideration, we decided to honor her earnest request and administered a third course of IVIG to the patient, mirroring previous dosages and duration. the patient exhibited commendable tolerance throughout the 3 IVIG courses, marked by the absence of any adverse reactions. To our amazement, the patient’s symptoms once again exhibited dramatic improvement during the course of treatment. On the third day of the third IVIG session, the patient’s fever subsided (Fig. 3). After treatment, the patient was able to remain seated, communicate with a few words, and retain intact orientation but exhibited impaired cognitive abilities, particularly in numeracy skills.

**Figure 4.**
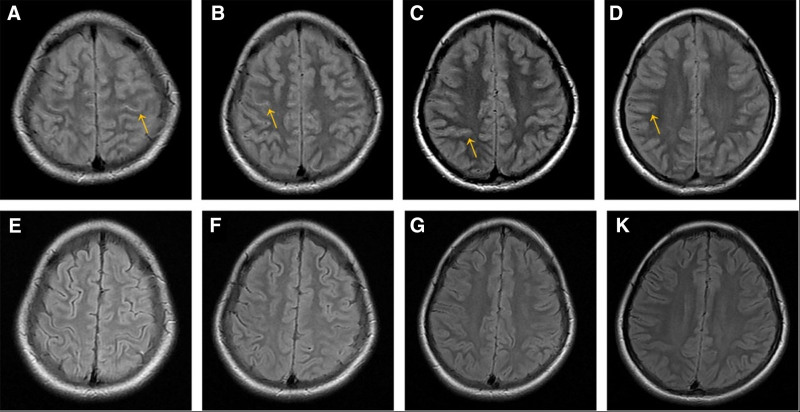
Magnetic resonance imaging findings. First day of admission, brain MRI T2-weighted fluid attenuated inversion recovery sequences revealed prominent linear signals (A–D). However, at discharge, 50th day of hospitalization, the majority of the linear signals seen in head MRI T2 sequences were resolved (E–K). MRI = magnetic resonance imaging.

Over the subsequent 2 weeks, the patient participated in rehabilitation therapy, gradually regaining strength and attempting to stand and walk. After this period, only nocturnal sleep disturbances and cognitive impairment persisted. Overall, appropriate responses were noted, although cognitive function remained impaired, as evidenced by the mRS score of 3. To further assess the cognitive impairments, the Mini-Mental State Examination (MMSE) and the Montreal Cognitive Assessment (MoCA) were conducted. The MMSE^[18]^ is a 30-point questionnaire widely used in clinical and research settings to assess cognitive impairment. It evaluates several cognitive domains, including registration (repeating named prompts), attention and calculation, recall, language, ability to follow simple commands, and orientation. The young girl’s MMSE score was 15, primarily reflecting impairments in orientation, attention and calculation, and recall. The MoCA^[19]^ is another widely used tool for assessing cognitive impairment, designed initially for detecting mild cognitive impairment (MCI) but later adopted in various clinical settings. With a score range from 0 to 30, it assesses multiple cognitive domains, including short-term memory, executive function, attention, and visuospatial abilities. The young girl’s MoCA score was 10, indicating significant impairment. The MoCA further highlighted impairments in language, visuospatial abilities, and executive function, in addition to the deficits noted on the MMSE. Follow-up EEG revealed no significant abnormalities, and the head MRI showed signal recovery (Fig. 4). The patient was discharged.

During a telephone follow-up 3 months post-discharge, improvements in walking speed and dexterity were observed, with an MMSE score of 21, MoCA score of 18 and an mRS score of 3. Six months post-discharge, the patient resumed daily school activities, although learning and memory abilities remained impaired, reflected in an MMSE score of 26, a MoCA score of 22, and a modified mRS score of 2, highlighting significant recovery in both cognitive and functional domains.

## 3. Discussion

The prodromal phase of anti-NMDAR encephalitis often presents with low-grade fever and nonspecific headaches. In contrast, signs of increased intracranial pressure and cervical rigidity are rarely reported in patients.^[14]^ Our case illustrates a rare set of manifestations in NMDAR encephalitis, where, in addition to the typical prodromal symptoms, there is a persistent presence of meningeal symptoms such as neck stiffness, fever, and elevated cerebrospinal fluid pressure during the active phase of the disease.

The exact mechanisms underlying these manifestations in this case are not fully understood. Meningitis can temporarily disrupt the blood-brain barrier, allowing NMDAR-specific B cells to infiltrate the central nervous system (CNS).^[20,21]^ Another possible mechanism involves the direct activation of NMDA-reactive B cells within the CNS by meningitis, indicating a loss of immune tolerance to NMDA receptors.^[22]^ The persistent fever observed during the course of NMDAR encephalitis in this case may be associated with concurrent noninfectious aseptic meningitis and intrathecal antibody synthesis.^[23]^ However, meningitis often occurs in the presence of pathogenic infections, where pathogens invade the subarachnoid space by interacting with endothelial cells at the blood-cerebrospinal fluid barrier’s postcapillary venules. Antecedent infections may play a role in triggering autoimmune encephalitis, but this has been conclusively demonstrated only in herpes simplex virus encephalitis. In this case, pathogenetic tests yielded negative results, suggesting the possibility of an unidentified causative factor leading to noninfectious aseptic meningitis, which continues to affect the meninges, resulting in fever, neck rigidity, and related symptoms. These mechanisms may help explain the rarity of meningitis as the predominant manifestation in cases of anti-NMDAR encephalitis.

In cases of anti-NMDAR encephalitis without tumors, early treatment with first and second-line immunotherapies, followed by long-term immunotherapy, is typically recommended.^[22]^ While some studies, such as a Chinese cohort study, have explored the efficacy of repeated first-line immunotherapy in this condition and suggested favorable outcomes with intensive therapy,^[24,25]^ specific guidelines regarding the frequency and timing of IVIG administration are lacking.

IVIG, a first-line immunotherapy for NMDAR encephalitis, has proven efficacy in treating autoimmune and inflammatory diseases.^[10]^ Its effects involve multiple mechanisms, including Fc receptor blockade, complement inhibition, augmentation of regulatory T cells, modulation of cytokines and growth factors, acceleration of autoantibody clearance, and activation of regulatory macrophages.^[26–28]^

While there is no specific guidance on the timing of repeated IVIG therapy in anti-NMDAR encephalitis, insights from primary immunodeficiency diseases suggest that maintenance doses every 3 to 4 weeks or maintaining trough serum IgG levels between 500 and 800 mg/dL can yield positive outcomes. Maintaining physiologic trough IgG levels is generally preferred.^[29–31]^ In some cases, higher IgG levels, even exceeding 1000 mg/dL, show therapeutic benefits. However, detailed pharmacokinetic studies on repeated high-dose IVIG in autoimmune diseases are lacking. The clinical significance of measuring IgG levels in autoimmune diseases is not firmly established.^[32]^ In the context of anti-NMDAR encephalitis, future studies measuring IgG levels could guide the frequency and timing of IVIG administration. After extensive literature review and research, we opted for an individualized first-line treatment for this patient. The rationale for this decision is summarized below.

1. Rapid Therapeutic Response: IVIG has demonstrated its potential to swiftly manifest therapeutic benefits in acute immune system disorders. For instance, in conditions like Kawasaki disease^[33]^ and the acute phase of myasthenia gravis,^[34]^ significant improvements have been observed within 1 to 7 days of initiating treatment. In clinical practice, we have also encountered numerous patients who exhibit rapid responses to IVIG. Hence, we consider it reasonable to attribute the patient’s swift symptom improvement following IVIG administration to the drug’s efficacy.
2. Distinctive Clinical Presentation: The patient’s distinctive clinical presentation was marked by atypical symptoms and a poor response to alternative treatments preceding the initial IVIG administration. Before the initial IVIG administration, the patient had been ill for 33 days, with 19 days of impaired consciousness and no discernible clinical improvement from previously administered medications. While our observation of hormonal effects may be brief, we noted a significant improvement in the initial days post the first IVIG administration, followed by relative symptom stabilization over the subsequent 2 to 3 days. While prolonged observation might reveal further symptom amelioration, the patient remained in a critical state. Recognizing that the duration of critical illness can significantly impact the patient’s prognosis, we aimed to minimize this critical phase. During the second and third doses, we once again observed a fluctuating reduction in the patient’s symptoms during the mid-dosing period, confirming this conjecture.
3. Safety of Shortening the Dosing Interval: This problem was addressed through an extensive literature review. Some autoimmune diseases, such as primary immune thrombocytopenia, recurrent miscarriage, and autoimmune dementia,^[35–37]^ recommend weekly IVIG repetition over several weeks as part of their treatment protocols. We deemed weekly IVIG repetition in the second week to be safe and rational.
4. Assessment of Medication Risk: We closely monitored the patient’s symptoms and physiological indicators during the first and second IVIG administrations, and no relevant risks were observed. Initial evaluation indicated good tolerability, which was further confirmed by subsequent treatment. Taking into account the patient’s responsiveness to IVIG, the safety and risk aspects of medication, and extensive communication with the family, supported by unwavering family backing, we decided to replicate this treatment plan in the third week.

In conclusion, our experience with individualized first-line therapy, including corticosteroids and repeated IVIG, for anti-NMDAR encephalitis highlights potential benefits but also emphasizes the need for further validation and cautious monitoring in the evolving treatment landscape. While the patient’s exceptional responsiveness to medication is notable, careful consideration and monitoring are advised regarding the repeated use of 3 courses of IVIG due to potential risks. Our approach aims to optimize treatment response and prognosis, considering various factors such as clinical presentation, safety concerns, and insights from existing literature on IVIG repetition in specific autoimmune conditions.

## Acknowledgments

The authors thank the patient for the sample contribution.

## Author contributions

**Supervision:** Jianhua Zhuang, Jin Xu.

**Writing – original draft:** Xin Jin.

**Writing – review & editing:** Xin Jin, Jianhua Zhuang, Jin Xu.

## References

[1] Dalmau J Tuzun E Wu HY. Paraneoplastic Anti-N-Methyl-D-Aspartate receptor encephalitis associated with ovarian teratoma. Ann Neurol. 2007;61:25–36. 17262855 10.1002/ana.21050 PMC2430743

[2] Dalmau J Lancaster E Martinez-Hernandez E Rosenfeld MR Balice-Gordon R. Clinical experience and laboratory investigations in patients with anti-NMDAR encephalitis. Lancet Neurol. 2011;10:63–74. 21163445 10.1016/S1474-4422(10)70253-2 PMC3158385

[3] Wang H Xiao Z. Current progress on assessing the prognosis for anti-N-methyl-D-aspartate receptor (NMDAR) encephalitis. Biomed Res Int. 2020;2020:7506590. 32352007 10.1155/2020/7506590 PMC7178504

[4] Lynch DR Rattelle A Dong YN Roslin K Gleichman AJ Panzer JA. Anti-NMDA receptor encephalitis: clinical features and basic mechanisms. Adv Pharmacol. 2018;82:235–60. 29413523 10.1016/bs.apha.2017.08.005

[5] Vyklicky V Korinek M Smejkalova T. Structure, function, and pharmacology of NMDA receptor channels. Physiol Res. 2014;63(Suppl 1):S191–203. 24564659 10.33549/physiolres.932678

[6] Amedonu E Brenker C Barman S. An assay to determine mechanisms of rapid autoantibody-induced neurotransmitter receptor endocytosis and vesicular trafficking in autoimmune encephalitis. Front Neurol. 2019;10:178. 30881339 10.3389/fneur.2019.00178 PMC6405626

[7] Nissen MS Ryding M Meyer M Blaabjerg M. Autoimmune encephalitis: current knowledge on subtypes, disease mechanisms and treatment. CNS Neurol Disord Drug Targets. 2020;19:584–98. 32640967 10.2174/1871527319666200708133103

[8] Delangle R Demeret S Canlorbe G. Anti-NMDA receptor encephalitis associated with ovarian tumor: the gynecologist point of view. Arch Gynecol Obstet. 2020;302:315–20. 32556515 10.1007/s00404-020-05645-9

[9] Graus F Titulaer MJ Balu R. A clinical approach to diagnosis of autoimmune encephalitis. Lancet Neurol. 2016;15:391–404. 26906964 10.1016/S1474-4422(15)00401-9 PMC5066574

[10] Titulaer MJ McCracken L Gabilondo I. Treatment and prognostic factors for long-term outcome in patients with anti-NMDA receptor encephalitis: an observational cohort study. Lancet Neurol. 2013;12:157–65. 23290630 10.1016/S1474-4422(12)70310-1 PMC3563251

[11] Florance NR Davis RL Lam C. Anti-N-methyl-D-aspartate receptor (NMDAR) encephalitis in children and adolescents. Ann Neurol. 2009;66:11–8. 19670433 10.1002/ana.21756 PMC2826225

[12] Suppiej A Nosadini M Zuliani L. Plasma exchange in pediatric anti-NMDAR encephalitis: a systematic review. Brain Dev. 2016;38:613–22. 26926399 10.1016/j.braindev.2016.01.009

[13] Dalmau J Armangué T Planagumà J. An update on anti-NMDA receptor encephalitis for neurologists and psychiatrists: mechanisms and models. Lancet Neurol. 2019;18:1045–57. 31326280 10.1016/S1474-4422(19)30244-3

[14] Stavrou M Yeo JM Slater AD Koch O Irani S Foley P. Case report: meningitis as a presenting feature of anti-NMDA receptor encephalitis. BMC Infect Dis. 2020;20:21. 31910823 10.1186/s12879-020-4761-1 PMC6947964

[15] Nosadini M Eyre M Molteni E; International NMDAR Antibody Encephalitis Consensus Group. Use and safety of immunotherapeutic management of N-methyl-D-aspartate receptor antibody encephalitis: a meta-analysis. JAMA Neurol. 2021;78:1333–44. 34542573 10.1001/jamaneurol.2021.3188 PMC8453367

[16] Rankin J. Cerebral vascular accidents in patients over the age of 60. II. Prognosis. Scott Med J. 1957;2:200–15. 13432835 10.1177/003693305700200504

[17] Teasdale G Jennett B. Assessment of coma and impaired consciousness. a practical scale. Lancet. 1974;2:81–4. 4136544 10.1016/s0140-6736(74)91639-0

[18] Pangman VC Sloan J Guse L. An examination of psychometric properties of the mini-mental state examination and the standardized mini-mental state examination: implications for clinical practice. Appl Nurs Res. 2000;13:209–13. 11078787 10.1053/apnr.2000.9231

[19] Asreddine ZS Phillips NA Bédirian V. The montreal cognitive assessment, MoCA: a brief screening tool for mild cognitive impairment. J Am Geriatr Soc. 2005;53:695–9. 15817019 10.1111/j.1532-5415.2005.53221.x

[20] Irani SR Bera K Waters P. N-Methyl-D-Aspartate antibody encephalitis: temporal progression of clinical and paraclinical observations in a predominantly non-paraneoplastic disorder of both sexes. Brain. 2010;133(Pt 6):1655–67. 20511282 10.1093/brain/awq113 PMC2877907

[21] Dalmau J Gleichman AJ Hughes EG. Anti-NMDA-receptor encephalitis: case series and analysis of the effects of antibodies. Lancet Neurol. 2008;7:1091–8. 18851928 10.1016/S1474-4422(08)70224-2 PMC2607118

[22] Liu Y Tian Y Guo R. Anti-NMDA receptor encephalitis: retrospective analysis of 15 cases, literature review, and implications for gynecologists. J Healthc Eng. 2022;2022:4299791. 35340259 10.1155/2022/4299791 PMC8941556

[23] Tominaga N Kanazawa N Kaneko A. Prodromal headache in Anti-NMDAR encephalitis: an epiphenomenon of NMDAR autoimmunity. Brain Behav. 2018;8:e01012. 29856136 10.1002/brb3.1012 PMC6043713

[24] Xu X Lu Q Huang Y. Anti-NMDAR encephalitis: a single-center, longitudinal study in China. Neurol Neuroimmunol Neuroinflamm. 2019;7:e633. 31619447 10.1212/NXI.0000000000000633 PMC6857906

[25] Hu S Lan T Bai R Jiang S Cai J Ren L. HSV encephalitis triggered anti-NMDAR encephalitis: a case report. Neurol Sci. 2021;42:857–61. 33420613 10.1007/s10072-020-04785-9

[26] Anthony RM Kobayashi T Wermeling F Ravetch JV. Intravenous gammaglobulin suppresses inflammation through a novel T(H)2 pathway. Nature. 2011;475:110–3. 21685887 10.1038/nature10134 PMC3694429

[27] Gelfand EW. Intravenous immune globulin in autoimmune and inflammatory diseases. N Engl J Med. 2012;367:2015–25. 23171098 10.1056/NEJMra1009433

[28] Othy S Hegde P Topcu S. Intravenous gammaglobulin inhibits encephalitogenic potential of pathogenic T cells and interferes with their trafficking to the central nervous system, implicating sphingosine-1 phosphate receptor 1-mammalian target of rapamycin axis. J Immunol. 2013;190:4535–41. 23526819 10.4049/jimmunol.1201965

[29] Berger M. Incidence of infection is inversely related to steady-state. (Trough) serum IgG level in studies of subcutaneous IgG in PIDD. J Clin Immunol. 2011;31:924–6. 21643892 10.1007/s10875-011-9546-2

[30] Perez EE Orange JS Bonilla F. Update on the use of immunoglobulin in human disease: a review of evidence. J Allergy Clin Immunol. 2017;139:S1–S46. 28041678 10.1016/j.jaci.2016.09.023

[31] Orange JS Grossman WJ Navickis RJ Wilkes MM. Impact of trough IgG on pneumonia incidence in primary immunodeficiency: a meta-analysis of clinical studies. Clin Immunol. 2010;137:21–30. 20675197 10.1016/j.clim.2010.06.012

[32] Chapel HM Lee M. Immunoglobulin replacement in patients with chronic lymphocytic leukemia (CLL): kinetics of immunoglobulin metabolism. J Clin Immunol. 1992;12:17–20. 1551938 10.1007/BF00918268

[33] Agarwal S Agrawal DK. Kawasaki disease: etiopathogenesis and novel treatment strategies. Expert Rev Clin Immunol. 2017;13:247–58. 27590181 10.1080/1744666X.2017.1232165 PMC5542821

[34] Zinman L Bril V. Ivig treatment for myasthenia gravis: effectiveness, limitations, and novel therapeutic strategies. Ann N Y Acad Sci. 2008;1132:264–70. 18567877 10.1196/annals.1405.038

[35] Kado R McCune WJ. Treatment of primary and secondary immune thrombocytopenia. Curr Opin Rheumatol. 2019;31:213–22. 30920453 10.1097/BOR.0000000000000599

[36] Carp H. Immunotherapy for recurrent pregnancy loss. Best Pract Res Clin Obstet Gynaecol. 2019;60:77–86. 31521575 10.1016/j.bpobgyn.2019.07.005

[37] Sechi E Flanagan EP. Diagnosis and management of autoimmune dementia. Curr Treat Options Neurol. 2019;21:11. 30809732 10.1007/s11940-019-0550-9

